# Carotid plaque thickness predicts cardiovascular events and death in patients with chronic kidney disease

**DOI:** 10.1186/s12882-024-03831-4

**Published:** 2024-10-31

**Authors:** Sasha S. Bjergfelt, Ida M. H. Sørensen, Laerke Urbak, Klaus F. Kofoed, Theis Lange, Bo Feldt-Rasmussen, Henrik Sillesen, Christina Christoffersen, Susanne Bro

**Affiliations:** 1 Department of Nephrology, Rigshospitalet, University of Copenhagen, Blegdamsvej 9, Copenhagen, DK-2100 Denmark; 2 Department of Biomedical Sciences, Faculty of Health and Medical Sciences, University of Copenhagen, Blegdamsvej 3B, Copenhagen, DK-2200 Denmark; 3 Department of Vascular Surgery, Rigshospitalet, University of Copenhagen, Blegdamsvej 9, Copenhagen, DK-2100 Denmark; 4 Department of Cardiology and Radiology, Rigshospitalet, University of Copenhagen, Blegdamsvej 9, Copenhagen, DK- 2100 Denmark; 5 Department of Clinical Medicine, Faculty of Health and Medical Sciences, University of Copenhagen, Blegdamsvej 3B, Copenhagen, DK-2200 Denmark; 6 Department of Public Health (Biostatistics), Faculty of Health and Medical Sciences, University of Copenhagen, Øster Farimagsgade 5, Copenhagen, DK-1014 Denmark; 7 Department of Clinical Biochemistry, Rigshospitalet, University of Copenhagen, Blegdamsvej 9, Copenhagen, DK- 2100 Denmark

**Keywords:** Chronic kidney disease, Cardiovascular risk, Cardiovascular events, MACE, Carotid ultrasound imaging, Maximal carotid plaque thickness, cPTmax, Plaque progression, Coronary artery calcium score, CACS

## Abstract

**Background:**

Classical risk scoring systems underestimate the risk of cardiovascular disease in chronic kidney disease (CKD). Coronary artery calcium score (CACS) has improved prediction of cardiovascular events in patients with CKD. The maximal carotid plaque thickness (cPTmax) measured in ultrasound scans of the carotid arteries has demonstrated similar predictive value as CACS in the general population. This is the first study to investigate whether cPTmax can predict cardiovascular events in CKD and to compare the predictive value of cPTmax and CACS in CKD.

**Method:**

Two hundred patients with CKD stage 3 from the Copenhagen CKD Cohort underwent ultrasound scanning of the carotid arteries. The assessment consisted of locating plaque and measuring the thickest part of the plaque, cPTmax. Based on the distribution of cPTmax, the participants were divided into 3 groups: No plaques, cPTmax 1.0–1.9 mm and cPTmax > 1.9 mm (median cPTmax = 1.9 mm among patients with plaques). To measure CACS, 175 of the patients underwent a non-contrast CT scan of the coronary arteries. The follow-up time spanned between the ultrasound scan and a predefined end-date or the time of first event, defined as a composite of major cardiovascular events or death of any cause (MACE).

**Results:**

The median follow-up time was 5.4 years during which 45 patients (22.5%) developed MACE. In a Cox-regression adjusted for classical cardiovascular risk factors, patients with cPTmax > 1.9 mm had a significantly increased hazard ratio of MACE (HR 3.2, CI: 1.1–9.3), *p* = 0.031) compared to patients without plaques. C-statistics was used to evaluate models for predicting MACE. The improvement in C-statistics was similar for the two models including classical cardiovascular risk factors plus cPTmax (0.247, CI: 0.181–0.312) and CACS (0.243, CI: 0.172–0.315), respectively, when compared to a model only controlled for time since baseline (a Cox model with no covariates).

**Conclusion:**

Our results indicate that cPTmax may be useful for predicting MACE in CKD. cPTmax and CACS showed similar ability to predict MACE.

**Supplementary Information:**

The online version contains supplementary material available at 10.1186/s12882-024-03831-4.

## Introduction

Chronic kidney disease (CKD) affects approximately 13.4% of the global population [1], and the prevalence is increasing with an ageing population and a growing number of people affected by diabetes, hypertension, and obesity [1–3]. Even mild CKD is closely associated with an increased risk of cardiovascular disease, which is the most important cause of morbidity and mortality in CKD [2, 4]. Cardiovascular disease is often asymptomatic and highly underestimated by the classical risk scoring systems in patients with CKD [5, 6]. However, assessment of asymptomatic vascular disease using coronary artery calcium score (CACS) has improved prediction of cardiovascular events in patients with CKD beyond the classical cardiovascular risk factors [7–10]. Also, a few prospective ultrasound studies of the carotid arteries indicate that the presence of carotid plaques may be useful in predicting cardiovascular events in CKD [11, 12].

The maximal carotid plaque thickness (cPTmax) is a simple ultrasound measure that has improved prediction of cardiovascular events similar to CACS in a non-CKD population [13]. We have previously demonstrated that cPTmax is increased in patients with CKD stage 3 compared with controls and closely associated with prevalent cardiovascular disease and calcification of the carotid and coronary arteries [14]. This is the first study to examine whether cPTmax can be used to predict major cardiovascular events and all-cause mortality (MACE) in patients with CKD. A secondary aim was to measure progression of cPTmax, and a tertiary aim was to compare the potential of cPTmax and CACS as predictors of MACE.

## Methods

### Study population

The cohort of the carotid ultrasound study has previously been described [14]. It consisted of a subgroup of patients with CKD stage 3 from the Copenhagen CKD Cohort (CPH CKD Cohort), a prospective, observational study investigating cardiovascular risk and imaging methods for early detection of cardiovascular disease in patients with CKD [15]. Patients were recruited consecutively between October 2015 and June 2017 from the nephrology outpatient clinic at Rigshospitalet, Copenhagen University Hospital. Inclusion criteria were age 30–75 years and CKD stages 1–5 (no dialysis). Exclusion criteria were previous renal transplantation with a functioning graft, active malignancy, pregnancy, and patients with intellectual disability, dementia, or psychosis. Among included patients (*n* = 741), those with CKD stage 3 were contacted and included consecutively in the carotid ultrasound study until 200 were enrolled.

The study followed the principles of the Declaration of Helsinki II and was approved by the Danish Scientific Ethical Committee (H-3-2011-069) and the Danish Data Protection Agency (30–0840). All participants signed a written informed consent before inclusion.

### Clinical data and biochemistry

Plasma and urine analytes were measured with a Cobas modular analyzer (Roche) using reagents from Roche Diagnostics. The EPI-CKD equation [16] was used to determine the estimated glomerular filtration rate (eGFR) and corresponding CKD stages at baseline as defined by the KDIQO guidelines [17].

As described previously, clinical, and demographic data at baseline were retrieved from electronic patient files and in-person interviews [14, 15]. Anthropometric and blood pressure measurements were collected during a physical examination. Participants were considered hypertensive if systolic blood pressure was > 140 mmHg and/or diastolic blood pressure was > 90 mmHg, or if participants were taking oral antihypertensive medication [15]. Hypercholesterolemia was defined as low-density lipoprotein (LDL) cholesterol > 3.0 mmol/l or treatment with cholesterol-lowering medication [15].

Diagnosis of diabetes mellitus (DM), type 1 or type 2, and cardiovascular disease at baseline was obtained through review of medical records. Cardiovascular disease was defined as a composite of prevalent coronary artery disease (a history of myocardial infarction, coronary artery angioplasty, stenting and/or coronary bypass surgery), previous cerebrovascular infarction, carotid endarterectomy or stenting, and/or peripheral artery disease (defined as a history of non-traumatic lower limb amputation, lower limb artery bypass surgery and/or angioplasty and/or stenting) [10]. Some patients had several cardiovascular diagnoses.

### Maximal carotid plaque thickness

A single operator (SSB) performed all the carotid artery ultrasound examinations with a Philips EPIQ 7 C ultrasound system equipped with a L12-3 transducer (48 Hz) and assessed plaque thickness using a Dicom viewer (Micro Dicom). The scanning protocol has previously been described in detail. In brief, the assessment was done by evaluation of consecutive, cross sectional images identifying the location with the greatest carotid plaque thickness (cPTmax), defined as the radial distance from the media–adventitia interface to the intima–lumen interface towards the centre of the arterial lumen [13]. Carotid plaque was defined as a focal structure encroaching into the arterial lumen of at least 0.5 mm, or 50% of the surrounding intima-media thickness value; or demonstrating a thickness ≥ 1.5 mm, as measured from the media-adventitia interface to the intima-lumen interface [18, 19]. For the statistical analysis only the anatomical side with the highest cPTmax was used. Images were analysed without knowledge of clinical data. As previously described, the intra-observer coefficient of variation (CV) was 9.3% [14]. The inter-observer agreement was assessed by SSB and LU, who independent of each other measured cPTmax from 50 ultrasound videos produced in a different cohort [20]. The correlation between the two sets of measurements was excellent with a correlation coefficient of 0.946 (CI: 0.902–0.970). Based on the distribution of cPTmax in the baseline ultrasound study, the individuals were divided into 3 groups: No plaques, cPTmax 1.0–1.9 mm and cPTmax > 1.9 mm (the median cPTmax among patients with plaques in the CKD group was 1.9 mm) [14].

### Progression of maximal carotid plaque thickness

All participants from the baseline carotid ultrasound study were offered a re-examination at the time of the follow-up study. The inter-scan period was defined as the length of time from the baseline ultrasound examination until the follow-up ultrasound examination. For analysis of progression of cPTmax, the baseline videos were re-analyzed intermixed with the follow-up videos. Based on the intra-observer CV of 9.3% when measuring cPTmax, progression was defined as an increase of cPTmax above 10.0%. In the statistical analyses, the progression rate (mm/year) was used to compensate for the inter-individual difference in time to re-scan.

### Arterial calcification

At baseline, a non-contrast 320-multidetector CT scan (Aquillon One, Toshiba medical Systems, Japan) was used to assess arterial calcification of the coronary arteries. Study methods and anatomical definitions have been described in detail [15]. Images were analysed without knowledge of clinical data. Calcium scoring was performed according to the Agatston method [21], and participants were divided into four calcium score categories: 0 (no calcification), 1-100, 101–400, > 400 [22]. Images of inadequate quality were excluded [10, 15].

### Study outcomes

We defined the primary endpoint, MACE, as a composite of cardiovascular events and all-cause mortality. The participants’ follow-up period began at the time of the baseline carotid ultrasound scan and ended at the time of study outcome onset (occurrence of a cardiovascular event or death), withdrawal, loss to follow-up, or the predefined end-date of the follow-up period (August 16th, 2022), whichever occurred first – meaning when several events occurred in the same patient, only time-to-first-event was considered in our analysis. Cardiovascular events were defined as previously described [10]: myocardial infarction, percutaneous coronary intervention, coronary bypass surgery, ischemic stroke, carotid endarterectomy or stenting, non-traumatic lower limb amputation, lower limb artery bypass graft, percutaneous transluminal angioplasty of a lower limb. All-cause mortality was defined as death from any cause, in-hospital or occurring outside of hospital. Outcome data were obtained through review of electronic medical records.

### Statistical analysis

Descriptive statistical analyses were performed using SPSS version 28.0.0.0 (IBM SPSS Statistics, New York,

USA). A value of *p <* 0.05 was considered statistically significant. Categorical variables are presented as n (%) and compared using the chi-square test. We used Fisher’s exact test in case of cells with an expected count < 5. Totals may not add up to 100% due to rounding. We used the Kolmogorov-Smirnov and Shapiro-Wilk tests to examine whether the continuous variables were normally distributed. If so, they are presented as mean ± standard error of the mean (SE) and analysed using the independent-samples t-test or Welch t-test depending on the heterogeneity of variances as assessed by Levene’s test for equality of variances. When comparing more than two groups, we used one-way ANOVA (or Welch’s ANOVA in case of heterogenicity). Skewed data are reported as median [interquartile range (IQR)] and compared using the Mann-Whitney U test or the Kruskal-Wallis test, when more than 2 groups were compared.

RStudio version 2022.12.0 was used for the survival analysis. To account for difference in follow-up length the Kaplan-Meier method was used to compare the risk of MACE between patients with: No carotid plaques at baseline, cPTmax 1.0–1.9 mm and cPTmax > 1.9 mm. Differences were compared using the log-rank tests for equality. We further applied a simple additive Cox proportional hazards regression analysis adjusted for the possible confounders: age, sex, hypertension, hypercholesterolemia, diabetes, and smoking (in pack years). The Likelihood ratio test was used to reject the hypothesis that eGFR and/or urine albumin/creatinine ratio influenced the model. Accordingly, eGFR and urine albumin/creatinine ratio was removed from the model. C-statistics was used to evaluate four models for predicting MACE: One with only classical cardiovascular risk factors, a second with cPTmax added to the classical cardiovascular risk factors, a third with CACS added to the classical cardiovascular risk factors and a fourth with both cPTmax and CACS added to the classical cardiovascular risk factors. All four models were compared to a model only controlled for time since baseline (in effect a Cox model with no covariates). In the C-statistics calculations, the CACS categories “CACS 1-100” and “CACS 101–400” were combined to secure that the measurements of cPTmax and CACS were divided into an equal number of categories.

## Results

### Baseline characteristics

Baseline characteristics of the 200 patients grouped according to the occurrence or non-occurrence of MACE are listed in Table 1. Compared to patients without MACE (*n* = 155), the patients with MACE (*n* = 45) were significantly older, most were men, they had a higher prevalence of diabetes (42.2% vs. 18.7%) and of previous cardiovascular disease (46.7% vs. 13.5%) and a higher number of pack years of smoking. Patients with MACE had a lower eGFR and a higher urine albumin/creatinine ratio. There was no difference in the prevalence of hypertension, or of hypercholesterolemia, but significantly more patients with MACE received lipid-lowering agents. Among the patients who experienced MACE, 86.7% had carotid plaques at baseline, in contrast to a plaque presence of 49.0% in the patients who did not develop MACE (*p* < 0.001). Also, the MACE group had a significantly higher baseline median cPTmax: 1.9 (1.5–2.8) mm versus 0 (0-1.6) mm in the non-MACE group (*p* < 0.001). Finally, patients with MACE had a significantly higher baseline median total CACS as compared with the non-MACE patients: 370 (87-1044) versus 7 (0-156), *p* < 0.001.

Accordingly, CACS > 400 was present in 45.9% of patients with MACE as compared with 15.2% of patients without MACE.

**Table 1:** Baseline characteristics of patients with and without MACE

Variable	Patients without MACE	Patients with MACE	*p*-value
Number of participants (n)	155	45	
Age (yr)	61 (52–70)	69 (65–72)	< 0.001
Female sex (n, %)	66 (42.6)	9 (20.0)	0.006
BMI	28.7 (± 0.5)	29.0 (± 0.9)	0.770
Hypertension (n, %)	138 (89.0)	40 (88.9)	0.978
Hypercholesterolemia (n, %)	124 (80.0)	39 (86.7)	0.346
Diabetes (n, %)	29 (18.7)	19 (42.2)	0.001
- Type 1 diabetes (n, %) - Type 2 diabetes (n, %)	4 (2.6) 25 (16.1)	2 (4.4) 17 (37.8)	
Prevalent CVD at baseline (n, %)	21 (13.5)	21 (46.7)	< 0.001
Smoking (pack years)	3 (0–20)	21 (0–50)	0.006
Medication (n, %)			
- Anti-hypertensive - Lipid-lowering - Diabetic treatment - Anti-platelet - Anti-coagulation	130 (83.9) 73 (47.1) 22 (14.2) 36 (23.2) 5 (3.2)	38 (84.4) 31 (68.9) 17 (37.8) 26 (57.8) 4 (8.9)	0.926 0.010 < 0.001 < 0.001 0.117
P-creatinine (µmol/l)	138 (120–163)	150 (132–169)	0.048
eGFR (ml/min/1.73m^2^)	41 (35–50)	38 (34–44)	0.039
P-HDL-cholesterol (mmol/l)	1.53 (± 0.05)	1.46 (± 0.09)	0.460
P-LDL-cholesterol (mmol/l)	3.0 (± 0.1)	2.7 (± 0.1)	0.073
P-triglycerides (mmol/l)	1.51 (1.12–2.24)	1.63 (1.28–2.51)	0.318
P-glucose (mmol/l)	6.1 (± 0.1)	6.9 (± 0.3)	0.017
P-ionized calcium (mmol/l)	1.23 (± 0.00)	1.20 (± 0.01)	0.003
P-phosphate (mmol/l)	1.02 (± 0.02)	1.05 (± 0.03)	0.454
P-urate (mmol/l)	0.45 (± 0.01)	0.44 (± 0.02)	0.800
Urine albumin/creatinine ratio x 10^− 3^	56 (7-271)	138 (35–692)	0.016
Plaque presence at baseline (n, %)	76 (49.0)	39 (86.7)	< 0.001
cPTmax at baseline (mm)	0 (0-1.6)	1.9 (1.5–2.8)	< 0.001
CACS	7 (0-156)	370 (87-1044)	< 0.001
CACS categories (n, %):			< 0.001
No coronary calcification CACS 1-100 CACS 101–400 CACS > 400	66 (47.8) 30 (21.7) 21 (15.2) 21 (15.2)	7 (18.9) 3 (8.1) 10 (27.0) 17 (45.9)	

BMI: body mass index. CVD: cardiovascular disease. cPTmax: maximal carotid plaque thickness

CACS: Coronary artery calcium score. Normally distributed data are presented as means ± (SE). Skewed data are reported as median (IQR). Categorical variables are presented as n (%). There were 1–2 missing values of the following variables: P-HDL-cholesterol, P-LDL-cholesterol, P-triglycerides, hypercholesterolemia, and alcohol intake, 9 missing values of the variable urine albumin/creatinine ratio and 25 missing values for CACS and CACS categories (missing values from 17 patients without MACE and 8 patients with MACE)

### Clinical outcomes

The patients were followed for an average of 5.4 years (1957 days). Twenty patients (10.0%) experienced a cardiovascular event (3 cases of myocardial infarction, 4 percutaneous coronary interventions, 2 coronary bypass grafts, 9 ischemic strokes, 1 endarterectomy, 5 cases of percutaneous transluminal angioplasty of a lower limb, 1 lower limb artery bypass graft, 3 non-traumatic lower limb amputations; patients may have had > 1 event) and 28 patients (14.0%) died during the follow-up period, leading to 45 endpoints for analysis (time-to-first event). Of note, during the follow-up period 14 patients started on chronic dialysis, 9 of these developed MACE. Two patients received a kidney transplant, none of these developed MACE.

### cPTmax is an independent predictor of MACE

**Fig. 1:**
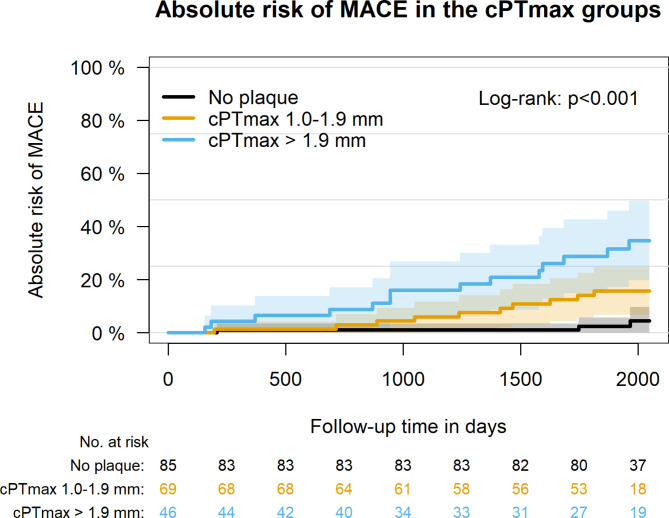
Absolute risk of MACE according to cPTmax groups. cPTmax: maximal carotid plaque thickness. MACE is a composite of cardiovascular events and death of all-causes.

In a crude absolute risk plot (Fig. 1), patients with no carotid plaques at baseline showed the lowest risk of MACE, whereas patients with cPTmax 1.0–1.9 mm showed an intermediate risk, and patients with cPTmax > 1.9 mm the highest risk (log-rank test, *p* < 0.001).

.

When using the group of patients with no plaques at baseline as the reference in an unadjusted Cox-regression analysis (Table 2), the hazard ratio (HR) of MACE was significantly increased in patients with cPTmax = 1.0–1.9 mm (HR = 3.8 (CI: 1.5–9.9), *p* = 0.004) and in patients with cPTmax > 1.9 mm (HR = 8.4 (CI: 3.4–20.8), *p* < 0.001). After adjustment for age, sex, diabetes, smoking, hypertension, and hypercholesterolemia, however, only patients with cPTmax > 1.9 mm showed a significantly increased HR of MACE (HR 3.2, (CI: 1.1–9.3), *p* = 0.031).

**Table 2:** Hazard ratios (HR) of MACE from univariate and multivariate Cox-regression analyses according to cPTmax groups or CACS categories

Covariate			HR	Confidence interval	*p*-value
	*Unadjusted*				
No carotid plaques			1.0 (Reference)		
cPTmax 1.0–1.9 mm			3.8	1.5–9.9	0.004
cPTmax > 1.9 mm			8.4	3.4–20.8	< 0.001
No coronary calcification			1.0 (Reference)		
CACS 1-100			0.9	0.2–3.7	0.940
CACS 101–400			3.9	1.5–10.3	0.006
CACS > 400			6.1	2.5–14.7	< 0.001
	*Adjusted*				
No carotid plaques			1.0 (Reference)		
cPTmax 1.0–1.9 mm			1.8	0.6–5.2	0.258
cPTmax > 1.9 mm			3.2	1.1–9.3	0.031
No coronary calcification			1.0 (Reference)		
CACS 1-100			0.5	0.1–2.1	0.342
CACS 101–400			1.9	0.7–5.5	0.225
CACS > 400			2.4	0.8–6.9	0.106

CACS: coronary artery calcium score. cPTmax: maximal carotid plaque thickness

CACS data available from 175 patients, of which 37 had events. Cox-regression adjusted for age, sex, smoking (pack years), hypertension, hypercholesterolemia, and diabetes

### cPTmax and CACS show similar potential as predictors of MACE

None of the CACS categories had a significantly increased HR, when using the group of patients with no calcification at baseline as the reference in a Cox-regression analysis adjusted for the same classical cardiovascular risk factors as above (Table 2). A step-wise Cox regression analysis (Additional file 1) showed that statistical significance was not reached even when the number of adjustment factors was reduced.

To further evaluate the potential of cPTmax and CACS as predictors of MACE, C-statistics was used (Table 3). The improvement in C-statistics compared to a model just using follow-up time was almost the same for the two models using classical cardiovascular risk factors plus cPTmax and CACS, respectively. Both methods had a higher C-statistics than the model which was only adjusted for classical cardiovascular risk factors. The highest C-statistics was observed in a model including classical cardiovascular risk factors and both cPTmax and CACS, however, the improvement was minor.

**Table 3:** C-statistics and difference in C-statistics for Cox-regression models predicting MACE based on classical cardiovascular risk factors, cPTmax and CACS

Variable	C-statistic	∆ C-statistic from base-model (95% CI)	^a^∆ *p*-value
Classical CV risk factors	0.719	0.219 (0.146–0.292)	< 0.001
Classical CV risk factors + cPTmax	0.747	0.247 (0.181–0.312)	< 0.001
Classical CV risk factors + CACS	0.743	0.243 (0.172–0.315)	< 0.001
Classical CV risk factors + cPTmax and CACS	0.764	0.264 (0.200–0.329)	< 0.001

cPTmax: maximal carotid plaque thickness. CACS: coronary artery calcium score. CV: cardiovascular. Number of valid cases = 170. Classical CV risk factors: age, sex, smoking (pack years), hypertension, hypercholesterolemia, and diabetes. ^a^∆ p-values are derived from comparison with the base model that only controlled for time since baseline

### Progression of cPTmax

One hundred and forty-one of the patients (70.5%) agreed to participate in the follow-up ultrasound study.

Reasons for non-participation (*n* = 59): Death (*n* = 17), weakened by illness (*n* = 17), lack of time (*n* = 4), Covid-19 related issues (*n* = 6), contact not possible (*n* = 6), transportation problems (*n* = 2), other/unknown reasons (*n* = 7).

The median inter-scan period was 4.0 years. Seventy-four patients (52.5%) showed progression of cPTmax above 10.0%; the median progression rate was 0.05 (0–0.13) mm/year.

Progression of cPTmax was more common in patients with carotid plaques at baseline as compared to patients without plaques at baseline (67.6% versus 37.1%, *p* < 0.001) (Table 4). Accordingly, the progression rate was significantly higher among patients with carotid plaques at baseline compared to patients without plaques at baseline: 0.09 (0.02–0.20) versus 0 (0-0.09) mm/year, *p* = 0.002 (Table 4).

Among the classical cardiovascular risk factors, only age was significantly associated with progression of cPTmax in a binary logistic regression analysis (OR: 1.062, *p* < 0.001) (Additional file 2).

**Table 4:** Progression of cPTmax according to plaque presence at baseline

	No plaques at baseline	Plaques at baseline	*p*-value
Number of patients	70	71	
Patients with progression (n,%)	26 (37.1)	48 (67.6)	< 0.001
Progression rate (mm/year)	0.00 (0.00-0.09)	0.09 (0.02–0.20)	0.002

Categorical variables are presented as n (%). Skewed data are reported as median (IQR)

## Discussion

CKD is closely associated with an increased risk of cardiovascular disease [2, 4] and the risk is highly underestimated by the classical risk scoring systems [5, 6]. This study is the first to show that a simple ultrasound measure, cPTmax, with a “risk threshold” of 1.9 mm is an independent predictor of cardiovascular events and death in patients with CKD stage 3. It is also the first study to measure progression of cPTmax. Progression was most pronounced in patients with carotid plaques present at baseline. Finally, the results indicate that cPTmax and CACS show similar ability to predict cardiovascular events and death in CKD.

The study by Sillesen et al. [13] showed that cPTmax predicted cardiovascular events similarly to the more complex carotid plaque burden in a large non-CKD population (*n* = 5808). In an adjusted model, they found a significantly increased risk of a composite event consisting of cardiovascular events and all-cause mortality in the participant groups with cPTmax > 1.84 mm. This is very similar to our finding of a “risk threshold” for cPTmax of > 1.9 mm. cPTmax was measured by the same method in the two studies, only the grouping of participants according to cPTmax was a little different.

So far, only few other studies using different methods for plaque quantification have addressed the predictive value of plaques for development of cardiovascular events and death in different stages of CKD [11, 12, 23]. Valdivielso et al. [11] showed that the number of carotid and femoral artery territories with plaques was an independent predictor of fatal and non-fatal cardiovascular events in patients with CKD stages 3–5 including patients on dialysis. Avramovski et al. [12] demonstrated that plaque scores based on the sum of plaque thickness in selected areas of the carotid or femoral arteries predicted cardiovascular death in patients on hemodialysis. In fact, the femoral plaque score was the strongest predictor.

Both the large study of the general population by Sillesen et al. [13] and the study of a population with CKD stages 3–5 by Valdvielso et al. [11] demonstrated that measurement by ultrasound of the unspecific carotid intima media thickness, which has previously been widely used as a measure of atherosclerosis, did not show any significance as a predictor of cardiovascular events. Recent recommendations are to use carotid plaque assessment instead of the more unspecific carotid intima-media thickness for cardiovascular risk prediction [19].

In the present study, progression of cPTmax was more common in patients with presence of carotid plaques at baseline as compared to patients without plaques at baseline. Accordingly, Gracia et al. [24] observed that CKD patients with no plaques at baseline were less likely to show progression after 24 months defined as an increase in the number of carotid and femoral artery territories showing a plaque. In about 40% of re-scanned patients in their study, progression was absent. As previously reported [14] the patients with plaques at baseline in our cohort were older, more were men and heavy smokers, they had a larger abdominal circumference, and a higher prevalence of diabetes and cardiovascular co-morbidity, as compared to patients without plaques at baseline. In our re-scan study with participation of 141 patients (70.5% of the baseline population), however, only age turned out to be significantly associated with progression. Gracia et al. (24) and Palanca et al. [25] in their larger studies found that both age, male sex, smoking and diabetes were associated with progression. Plaque progression was more likely to happen in patients with progression of CKD [24]. For CKD stage 3, Gracia et al. [24] also showed an association of progression with systolic blood pressure, plasma phosphate, use of phosphate binder and 25-hydroxy vitamin D. The last three factors are associated with bone and mineral disorder in CKD and are indicators of the uremic metabolic derangement, which might influence both arterial calcification and atherogenesis. Since follow-up data after the recent re-scan of our cohort are not available, we cannot yet evaluate whether the progression rate is a more sensitive risk predictor of cardiovascular events than baseline cPTmax measurement. Potentially, measurement of cPTmax progression could be used for evaluation of treatment interventions, in the same way the more extensive measurement, carotid plaque burden, has already been used [26–28].

Although not confirmed in the present small study, several studies including a recent study from the CPH CKD cohort, have proven CACS to be an independent predictor of cardiovascular disease and death in patients with CKD [7–10]. Especially the finding of CACS as a significant predictor of MACE in the larger study based on the same cohort (*n* = 570) [10] strongly indicates that the lack of a significant association between CACS and MACE in the present study was due to a lower number of patients and events. However, larger studies are needed to confirm the ability of both cPTmax and CACS to predict MACE in patients with all stages of CKD.

A large general population study demonstrated that cPTmax predicted cardiovascular events and all-cause mortality similarly to the carotid plaque burden estimates [13], which had a similar predictive ability as CACS [29]. The results of the present study indicate that cPTmax could also be used as a predictor of cardiovascular events and death in patients with CKD, at least in patients with CKD stage 1–3. The close correlation between cPTmax and CACS categories described in our previous paper based on the same cohort [14] and the similar improvement in C-statistics for cPTmax and CACS in the present study, further support that cPTmax is not inferior to CACS and may be a useful risk predictor in CKD. However, we cannot extrapolate our findings to patients with CKD stages 4–5. cPTmax is a measure of atherosclerosis in the carotid arteries, whereas CACS is a measure of intimal and medial calcification in the coronary arteries (the two forms are indistinguishable by CT-scans). Intimal calcification reflects atherosclerosis, which is highly prevalent in CKD and associated with ischemic heart disease, stroke and peripheral artery disease [30]. Medial calcification increases with higher CKD stages and leads to arterial stiffness resulting in high systolic blood pressure, left ventricular hypertrophy, heart failure and arrhythmias [30]. Thus, it is possible that cPTmax is a weaker predictor than CACS in more advanced CKD. Accordingly, a small study of patients on dialysis showed only a moderate association between CACS and carotid plaque burden [23].

Compared with CACS, ultrasound imaging is more convenient, more widely available, and without radiation exposure. cPTmax is a simple and highly reproducible measure of atherosclerosis when ultrasound examination and reading are performed by a trained examiner (high intra- and interobserver agreement). This underlines its potential for use as a cardiovascular risk predictor in future single- or multi-center studies and in the clinical setting.

### Strengths and weaknesses of this study

This study has several weaknesses. First, it is a relatively small study including only patients with CKD stage 3. Secondly, patients were included despite having had a cardiovascular event prior to inclusion. Third, the carotid plaque burden was not measured for comparison with cPTmax. Even though it has previously been shown that cPTmax has a similar predictive value as carotid plaque burden in the general population, we cannot know if this is true for CKD patients as well, considering that they often have a higher atherosclerotic burden compared with the general population. Moreover, patients with CKD develop both intimal and medial arterial disease. We did not measure cPTmax with the baseline and re-scan videos side by side, which means we cannot know, whether the two measurements were done on the same plaque. We only measured maximal plaque thickness in the carotid arteries. However, based on findings from other ultrasound studies [11, 12] it is possible that the combination of maximal plaque thickness from several arterial sites, e.g. both the carotid and femoral arteries could improve prediction of cardiovascular events in CKD.

One of the strengths of this study is that the same person performed all the ultrasound scans and did all the video analyses. The baseline measurements of cPTmax were done 4 years prior to follow-up and therefore the reader could not know which patients would develop MACE. A large part of the patients experienced MACE which improves the statistical strength in our survival analysis. Furthermore, the study population represents real-world CKD stage 3 patients very well because patients with prevalent cardiovascular disease and diabetes were included. A large part of the patients underwent both an ultrasound scan of the carotid arteries and a non-contrast CT-scan of the heart, which made it possible to compare the predictive value of cPTmax and CACS.

## Conclusion

In conclusion, our results indicate that measurement of cPTmax may be useful for prediction of cardiovascular events and death in patients with CKD, at least in CKD stages 1–3. Progression of cPTmax was most pronounced in patients with carotid plaques present at baseline. Further, the results indicate that cPTmax and CACS show similar ability to predict cardiovascular events and death in CKD. More and larger studies are needed to evaluate the validity of cPTmax as a predictor of cardiovascular disease in patients with all stages of CKD, and of cPTmax progression as a predictor of changes in risk.

Part of the results from this study has been presented as an oral presentation at the ERA-EDTA conference in Milano, Italy, 17.6.2023.

## Electronic supplementary material

Below is the link to the electronic supplementary material.

Supplementary Material 1

## Data Availability

The datasets generated during the current study are not publicly available due to Danish legal restrictions but available on reasonable request from The Steering Committee of the CPH CKD cohort project (secretary Christine.Korsholm.Nielsen@regionh.dk), provided relevant ethical and legal permissions have been attained priorly and researchers meet the criteria for access to confidential data.

## Abbreviations

BMI: Body mass index
CACS: Coronary artery calcium score
CKD: Chronic kidney disease
CPH: Copenhagen
cPTmax: Maximal carotid plaque thickness
CT: Computer tomography
CV: Coefficient of variation
CVD: Cardiovascular disease
DM: Diabetes mellitus
eGFR: Estimated glomerular filtration rate
HDL-C: High-density lipoprotein cholesterol
IQR: Interquartile range
LDL-C: Low-density lipoprotein cholesterol
MACE: A composite of cardiovascular events and all-cause mortality
n: Number
P: Plasma
SE: Standard error of the mean
yr: Years
