# Supplementary material for: Carotid plaque thickness predicts cardiovascular events and death in patients with chronic kidney disease
Source: BMC Nephrol. 2024 Oct 31;25:389. doi: 10.1186/s12882-024-03831-4 (PMC11526655; doi:10.1186/s12882-024-03831-4)
Supplement: Supplementary file 1 — Supplementary Material 1 [file 12882_2024_3831_MOESM1_ESM.docx]

**Supplementary information**

**Additional file 1 Supplementary Table 1**

**Hazard ratios (HR) of MACE from stepwise multivariate Cox-regression analyses according to cPTmax groups or CACS categories.**

| **Covariate** |  | **HR** | | **Confidence interval** | | **p-value** |
| --- | --- | --- | --- | --- | --- | --- |
| *Adjusted for age and sex* | | | | | | |
| No carotid plaques |  | 1.0 (Reference) | |  | |  |
| cPTmax 1.0-1.9 mm |  | 2.3 | | 0.8 – 6.1 | | 0.111 |
| cPTmax > 1.9 mm |  | 3.7 | | 1.3 – 10.5 | | 0.013 |
| No coronary calcification |  | 1.0 (Reference) | |  | |  |
| CACS 1-100 |  | 0.6 | | 0.2 – 2.5 | | 0.520 |
| CACS 101-400 |  | 2.3 | | 0.8 – 6.6 | | 0.108 |
| CACS > 400 |  | 2.7 | | 1.0 – 7.8 | | 0.062 |
| *Adjusted for age, sex and hypertension* | | | | | | |
| No carotid plaques |  | 1.0 (Reference) | |  | |  |
| cPTmax 1.0-1.9 mm |  | 2.3 | | 0.8 – 6.1 | | 0.109 |
| cPTmax > 1.9 mm |  | 3.8 | | 1.3 – 10.7 | | 0.012 |
| No coronary calcification |  | 1.0 (Reference) | |  | |  |
| CACS 1-100 |  | 0.6 | | 0.2 – 2.5 | | 0.521 |
| CACS 101-400 |  | 2.3 | | 0.8 – 6.6 | | 0.108 |
| CACS > 400 |  | 2.7 | | 1.0 – 7.8 | | 0.062 |
| *Adjusted for age, sex, hypertension and hypercholesterolemia* | | | | | | |
| No carotid plaques |  | | 1.0 (Reference) | |  |  |
| cPTmax 1.0-1.9 mm |  | | 2.3 | | 0.8 – 6.3 | 0.104 |
| cPTmax > 1.9 mm |  | | 3.8 | | 1.3 – 10.8 | 0.012 |
| No coronary calcification |  | | 1.0 (Reference) | |  |  |
| CACS 1-100 |  | | 0.7 | | 0.1 – 2.4 | 0.451 |
| CACS 101-400 |  | | 2.3 | | 0.8 – 6.6 | 0.110 |
| CACS > 400 |  | | 2.8 | | 1.0 – 8.1 | 0.054 |
| *Adjusted for age, sex, hypertension, hypercholesterolemia and smoking (pack years)* | | | | | | |
| No carotid plaques |  | 1.0 (Reference) | |  | |  |
| cPTmax 1.0-1.9 mm |  | 2.2 | | 0.8 – 6.1 | | 0.130 |
| cPTmax > 1.9 mm |  | 3.8 | | 1.3 – 10.8 | | 0.013 |
| No coronary calcification |  | 1.0 (Reference) | |  | |  |
| CACS 1-100 |  | 0.6 | | 0.1 – 2.3 | | 0.438 |
| CACS 101-400 |  | 2.3 | | 0.8 – 6.5 | | 0.115 |
| CACS > 400 |  | 2.9 | | 1.0 – 8.2 | | 0.051 |

CACS: coronary artery calcium score. cPTmax: maximal carotid plaque thickness.
CACS data available from 175 patients, of which 37 had events.

**Additional file 2 Supplementary Table 2**

**Binary logistic regression analysis examining the association of classical cardiovascular risk factors with progression of cPTmax.**

| Covariate | Odds ratio | p-value |
| --- | --- | --- |
| Age | 1.063 | < 0.001 |
| Male gender | 1.291 | 0.502 |
| Hypertension | 0.681 | 0.500 |
| Hypercholesterolemia | 1.312 | 0.565 |
| Diabetes | 1.194 | 0.705 |
| Smoking (pack years) | 1.007 | 0.423 |

Number of valid cases = 140.
